# Complete Chromosome Sequence of a Mycolactone-Producing Mycobacterium, *Mycobacterium pseudoshottsii*

**DOI:** 10.1128/genomeA.01363-17

**Published:** 2017-11-30

**Authors:** Mitsunori Yoshida, Yuji Miyamoto, Yoshitoshi Ogura, Tetsuya Hayashi, Yoshihiko Hoshino

**Affiliations:** a Department of Mycobacteriology, Leprosy Research Center, National Institute of Infectious Diseases, Higashimurayama, Tokyo, Japan; b Department of Bacteriology, Faculty of Medical Sciences, Kyushu University, Fukuoka, Japan

## Abstract

*Mycobacterium pseudoshottsii* is a fish pathogen that produces mycolactone. Here, we report the complete chromosome sequence of a type strain of *M. pseudoshottsii* (JCM 15466). The sequence will represent essential data for future phylogenetic and comparative genome studies of mycolactone-producing mycobacteria.

## GENOME ANNOUNCEMENT

A variety of mycobacteria can be fish pathogens, and species-associated host specificity is supposed to exist, but this specificity has not been fully elucidated (1). Among fish pathogens, *Mycobacterium marinum* is widely distributed around the world and is the most extensively studied. *Mycobacterium abscessus*, *Mycobacterium chelonae*, *Mycobacterium fortuitum*, *Mycobacterium salmoniphilum*, *Mycobacterium shottsii*, *Mycobacterium stephanolepidis*, and *Mycobacterium pseudoshottsii* are also commonly identified (1–6). It is also noteworthy that many of these have zoonotic potential. *M. pseudoshottsii* was originally isolated from wild striped bass (4), but its infection can occur in a wide range of farmed fishes (7). It produces mycolactone, as do its close relatives (*Mycobacterium liflandii*, *Mycobacterium shinshuense*, and *Mycobacterium ulcerans*, but not *M. marinum*) (8). Here, we report the complete chromosome sequence of *M. pseudoshottsii* JCM 15466^T^.

The strain was grown with Middlebrook 7H9 medium. DNA was extracted using the standard phenol-chloroform method. The chromosome sequence was determined using PacBio reads (71,975 subreads) obtained by an RSII system (Menlo Park, CA, USA). The reads were assembled with Canu version 1.5 and circularized using Circlator (9, 10). Illumina 150-bp × 2 paired-end reads (1,283,726 reads) were obtained by a MiSeq sequencer (Illumina, San Diego, CA, USA) and mapped to the assembly using the Burrows-Wheeler aligner (11) for sequence and assembly error correction with Pilon (12). Annotation was carried out with the DDBJ Fast Annotation and Submission Tool (DFAST) (https://dfast.nig.ac.jp/). Average nucleotide identity (ANI) was calculated by JSpeciesWS (13).

The chromosome of JCM 15466^T^ is 6,061,597 bp in length (65.6% G+C content). ANIs to related mycobacteria were 99.43% to *M. liflandii* (strain 128FXT), 99.24% to *M. shinshuense* (strain ShT-P), 99.14% to *M. ulcerans* (strain Agy99), and 98.99% to *M. marinum* (strain M), supporting the proposed taxonomic position of *M. pseudoshottsii* as a subspecies of *M. ulcerans* (14). The chromosome of *M. pseudoshottsii* is smaller than those of *M. marinum* strain M (6,636,827 bp) and *M. liflandii* 128FXT (6,208,955 bp) but larger than those of *M. shinshuense* ShT-P (5,899,681 bp) and *M. ulcerans* Agy99 (5,631,606 bp). The number of predicted protein-coding sequences on the chromosome (5,617) exceeds the reported numbers for *M. ulcerans* Agy99 (4,160), *M. liflandii* 128XFT (4,730), and *M. shinshuense* ShT-P (5,015) and is almost equivalent to that of *M. marinum* M (5,424) (15). The numbers of rRNA operons (3), tRNA genes (50), and clustered regularly interspaced short palindromic repeat (CRISPR) loci (3) are the same or almost equivalent to those of close relatives. We identified many pseudogenes (538), as observed for *M. shinshuense* ShT-P (451) and *M. ulcerans* Agy99 (771). Although the draft genome sequence of JCM 15466^T^ was previously determined (16), the numbers of predicted genes might differ from those obtained in this study. In addition, we identified 171 copies of insertion sequence (IS) elements on the chromosome, which were classified into 2 kinds of elements. The complete chromosome sequence of *M. pseudoshottsii* JCM 15466^T^ will represent essential data for future phylogenetic and comparative genome studies and for a better understanding of the evolution of mycolactone-producing mycobacteria.

### Accession number(s).

The chromosome sequence has been deposited at DDBJ/ENA/GenBank under the accession no. AP018410.
